# Genetic relationships between sweet cherry (*Prunus avium* L.) and sour cherry (*P. cerasus* L.) as revealed using fruit characterizations and chloroplast microsatellites

**DOI:** 10.1002/fsn3.3858

**Published:** 2023-11-23

**Authors:** Ali Khadivi, Somayeh Goodarzi, Mostafakamal Shams

**Affiliations:** ^1^ Department of Horticultural Sciences, Faculty of Agriculture and Natural Resources Arak University Arak Iran; ^2^ Department of Plant Physiology and Biotechnology, Faculty of Biology University of Gdansk Gdansk Poland

**Keywords:** breeding, cherries, chloroplast, fruit, variation

## Abstract

In the present study, the genetic diversity as well as the relationship between sweet cherry (*Prunus avium* L.) and sour cherry (*P. cerasus* L.) genotypes were investigated based on fruit traits and chloroplast microsatellites (cpSSRs). Analysis of variance showed that the studied genotypes have significant differences in the studied traits. In sweet cherries, the average fruit weight was 4.49 g with a coefficient of variation (CV) of 15.62%, the average stone weight was 0.34 g with a CV of 15.67%, and the average total soluble solids was 11.90% with a CV of 22.06%. Also, in sour cherries, the average fruit weight was 2.65 g with a coefficient of variation (CV) of 14.27%, the average stone weight was 0.28 g with a CV of 12.27%, and the average total soluble solids was 10.90% with a CV of 19.80%. Principal component analysis (PCA) showed that 83.80% of the observed variance was explained by the first three components. The cluster analysis separated genotypes of sweet and sour cherries and put them into two main groups. Four cpSSR primers produced distinct and different alleles among sweet and sour cherries. The cpSSR loci separated sweet and sour cherries from each other, which confirms the theory that chloroplast genome of sour cherry is not derived from sweet cherry. The present results provided new insights regarding the extent of diversity of individuals and also determined the relatedness and obtained information on genetic diversity of sweet and sour cherries.

## Graphical abstract

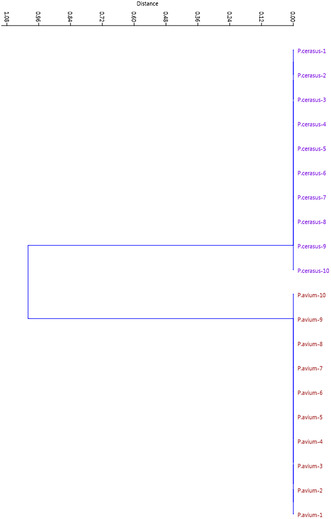

## 1 INTRODUCTION

Several types of classification based on morphological studies have been reported for subgenus *Cerasus* (Moreno & Manzano, 2002). The section that is more related to edible cherries is *Eucerasus* and it has been agreed by most taxonomic scientists that it includes *Prunus avium* L. (sweet cherry), *P. cerasus* L. (sour cherry), and *P. fruticosa* Pall. (ground cherry). All these species originate from Europe, North Africa, Afghanistan, Türkiye, and Iran (Webster & Looney, 1996).

Sweet cherry is commercially important and its wild trees are also used to produce wood or as a foundation. This species is tall, and its height in some trees reaches 20 m (Webster & Looney, 1996). This species is diploid (2n = 2x = 16, AA) and is distributed in temperate regions (Moreno & Manzano, 2002). Sweet cherries are cultivated in more than 40 countries that have temperate to subtropical regions, and Türkiye, Iran, and several countries from Europe and America can be mentioned as the main areas of its cultivation (FAO STAT, 2021).

Sour cherries are consumed fresh, juice, and jam. This species is allotetraploid (2n = 4x = 32, AAFF) and is a natural hybrid between sweet cherry (paternal parent) and ground cherry (maternal parent). Sour cherry is a storage tree whose height varies from 3 to 10 or 15 m (Webster & Looney, 1996). Türkiye, Russia, Ukraine, Poland, and Iran are the main areas of its cultivation (Ates & Ozturk, 2022; FAO STAT, 2021).

Determining the genetic diversity in plant materials is of great importance and is the first and fundamental step to identify, preserve, and maintain the genetic resources, which are considered the basis for genetic research and breeding programs. The genetic diversity of domesticated plants has been stabilized due to the use of limited genetic bases in breeding programs, and the diversity of native cultivars is also decreasing (Brown, 1978).

Morphological classification is a useful guide to identify species relationships and increases the knowledge of plant breeders and gene bank managers. Also, knowing the relationships between traits (regression and correlation relationships) can be useful for the development of new commercial cultivars and resistant and short bases (Hrotko et al., 2008).

The study of the chloroplast genome began in the 1950s when plant biologists first discovered that the chloroplast has its DNA (Sugiura, 2003). Chloroplast microsatellites (cpSSRs) have been used to investigate the phylogeny and diversity of chloroplasts in various fruit trees and have resolved many ambiguities. Brettin et al. (2000) reported that chloroplast inheritance in *Prunus* genus is maternal.

Chloroplast microsatellites (cpSSRs) for the genus *Prunus* have evolved (Brettin et al., 2000; Ohta et al., 2005) and have been used to investigate the phylogenetic relationships of several species of the subgenus *Cerasus* (Ma et al., 2009; Ohta et al., 2006, 2007) and to evaluate phylogeny of several species of *Prunophora* subgenus (Decroocq et al., 2004; Xuan et al., 2010). Iran has rich cherry germplasm resources and therefore, categorizing and characterizing this gene pool is an essential step in the selection and breeding. The present study aimed to investigate the genetic diversity as well as the relationship between sweet cherry and sour cherry genotypes based on fruit traits and chloroplast microsatellites (cpSSRs). Characterization, evaluation, and documentation system for the studied germplasm will be invaluable for manipulating management of genetic conservation, production, and further breeding programs for sustainable improvement of cherries.

## 2 MATERIALS AND METHODS

### 2.1 Plant material

The genetic diversity as well as the relationship between 10 genotypes of sweet cherry and 10 genotypes of sour cherry were investigated based on fruit traits and chloroplast microsatellites (cpSSRs). The studied genotypes were collected from the Senejan area in Arak region, Markazi province, Iran (34°05′30″ N, 49°45′10″ E, 1708 m above sea level). The mean annual temperature and rainfall in the Arak region is 13.80°C and 320 mm, respectively. Different horticultural practices including fertilizer, application spraying, irrigation, and other cultural practices were made at regular intervals each year. The trees were mature, healthy, and in cropping condition.

### 2.2 Morphological evaluations

Fruits were hand‐harvested randomly from different parts of the trees. The time of fruit harvesting was determined based on the ripening time, which included color changes, appearance, and taste of the fruits. Ten quantitative traits related to fruit were measured. The traits related to fruit size, such as length, diameter, and width were measured using a digital caliper (P1026‐500‐200 model, Pride, China). Fruit weight, fruit stalk weight, and stone weight were measured using an electronic scale (SILIT WMF model, WMF, Germany) with an accuracy of 0.01 g.

The main composition of dissolved total soluble solids (TSS) is sugars, and for this reason, a refractometer (pocket PAL‐1 ATAGO Corporation, Tokyo, Japan) was used to measure TSS (%). To measure the titratable acidity (TA), 5 mL of fruit juice was diluted with 95 mL of distilled water and titrated with 0.10 NaOH to pH = 8.20 using a pH meter (HANNA pH 212; Woonsocket, RI, USA), and then using the relevant formula, the amount of acid was calculated in terms of malic acid (predominant acid of cherries) in 100 cc of fruit juice. In calculating titratable acidity, the dominant acid of the fruit should be considered, and based on that, the desired coefficient (dominant acid equivalent) should be applied. This coefficient is 67 for malic acid (Webster & Looney, 1996).

The color index of fruit juice, which represents the anthocyanin content, was measured based on the absorbance of diluted fruit juice using a spectrophotometer (Perkin Elmer, Lambda EZ201, USA) at a wavelength of 510 nm (the average wavelength of maximum absorption of anthocyanins) and distilled water was used as a control for the device's zero.

### 2.3 Molecular evaluations

In the first stage, the young leaves of the studied genotypes were collected in April and June and kept in a freezer at −80°C. In the second step, the method of Doyle and Doyle (1987) was used for DNA extraction. The quantity and quality of the obtained DNA were determined using spectrophotometry and nanodrop methods at 260 and 280 nm wavelengths and DNA electrophoresis in agarose gel with a concentration of 1.00% and was prepared for PCR operation.

Chloroplast diversity and phylogenetic relationships of the studied genotypes were analyzed using four chloroplast SSR loci (cpSSRs). The PCR reaction was prepared in a volume of 20 μL, which contains 20 mM (mM) of Tris–HCl buffer with a pH equal to 8.4, 4 mM of MgCl_2_ solution, 0.1 mM of each dNTP; 0.2 μM of each primer, and 40 ng of DNA and 0.45 units (U) of *Taq* polymerase enzyme.

AB9700 thermocycler (Applied Biosystems) was used for PCR reaction. The thermal cycles of this protocol include an initial conditioning stage at 94°C for 2 min; 35 cycles at 94°C for 45 s; at a temperature of 57°C for 45 s; It was at a temperature of 72°C for 1 min and finally, at a temperature of 72°C for 5 min. PCR product electrophoresis was analyzed in MetaPhor Agarose gel with a concentration of 1.50%.

### 2.4 Statistical analysis

Variance analysis for all traits was done using SAS software (SAS Institute, 1990). Descriptive statistics, simple correlation between traits, cluster, and principal component analysis (PCA) based on factor rotation and Varimax method were performed using SPSS (Version 16.0) software (SPSS Inc., Chicago, IL, USA, Norusis, 1998). Cluster analysis was done using PAST software (Hammer et al., 2001).

## 3 RESULTS AND DISCUSSION

### 3.1 Morphological analysis

Analysis of variance showed that the studied genotypes have significant differences in the studied traits (not shown); therefore, all traits were used in the next stages of the analysis. In both sweet and sour cherries, the highest coefficient of variation (CV) was observed in anthocyanin index (44.42% and 43.48%, respectively). The lowest CV in sweet cherry was related to fruit length (8.09%) and in sour cherry, it was related to fruit diameter (4.06%) (Table 1).

**TABLE 1:** Statistical descriptive parameters for fruit traits used to study sweet and sour cherries.

No.	Trait	Unit	Sweet cherry					Sour cherry				
			Min	Max	Mean	SD	CV (%)	Min	Max	Mean	SD	CV (%)
V1	Fruit weight	g	3.50	5.47	4.49	0.70	15.62	2.15	3.58	2.65	0.38	14.27
V2	Stone weight	g	0.25	0.42	0.34	0.05	15.67	0.24	0.34	0.28	0.03	12.27
V3	Fruit length	mm	15.24	21.12	18.64	1.51	8.09	13.31	16.54	14.71	1.13	7.70
V4	Fruit width	mm	15.70	21.49	18.74	1.74	9.31	14.41	16.74	15.89	0.71	4.45
V5	Fruit diameter	mm	13.36	18.56	16.20	1.70	10.47	16.12	18.60	17.25	0.70	4.06
V6	Fruit stalk length	mm	23.35	52.40	39.64	7.72	19.48	38.03	50.59	47.18	4.20	8.89
V7	Fruit stalk weight	g	0.07	0.12	0.09	0.01	15.39	0.05	0.09	0.07	0.02	23.12
V8	Titratable acidity	%	3.40	5.50	4.15	0.63	15.31	1.15	2.98	2.41	0.68	28.42
V9	Total soluble solids	%	9.00	17.30	11.90	2.63	22.06	7.50	14.00	10.90	2.16	19.80
V10	Anthocyanin Index	OD 510 nm	0.08	0.44	0.23	0.10	44.42	0.09	1.00	0.72	0.31	43.48

In sweet cherries, the range of the traits was as follows: fruit weight: 3.50–5.47 g, stone weight: 0.25–0.42 g, fruit length: 15.24–21.12 mm, fruit width: 15.70–21.49 mm, fruit diameter: 13.36–18.56 mm, fruit stalk length: 23.35–52.40 mm, fruit stalk weight: 0.07–0.12 g, TA: 3.40%–5.50%, TSS: 9.00%–17.30%, and anthocyanin index: 0.08–0.44 OD 510 nm (Table 1).

In sour cherries, the range of the traits was as follows: fruit weight: 2.15–3.58 g, stone weight: 0.24–0.34 g, fruit length: 13.31–16.54 mm, fruit width: 14.41–16.74 mm, fruit diameter: 16.12–18.60 mm, fruit stalk length: 38.03–50.59 mm, fruit stalk weight: 0.05–0.09 g, TA: 1.15%–2.98%, TSS: 7.50%–14.00%, and anthocyanin index: 0.09–1.00 OD 510 nm (Table 1).

The total data of sweet and sour cherries were used for further analysis. Pairwise correlations between the recorded traits are shown in Table 2. Fruit weight showed positive and significant correlations with stone weight (*r* = .95), fruit length (*r* = .82), fruit width (*r* = .79), fruit stalk weight (*r* = .79), and TSS (*r* = .92), while it showed negative and significant correlation with fruit stalk length (*r* = .92).

**TABLE 2:** Simple correlations among the quantitative fruit variables utilized in the studied sweet and sour cherries.

No.	Trait	V1	V2	V3	V4	V5	V6	V7	V8	V9	V10
V1	Fruit weight	1									
V2	Stone weight	.86**	1								
V3	Fruit length	.94**	.83**	1							
V4	Fruit width	.91**	.89**	.92**	1						
V5	Fruit diameter	.85**	.84**	.83**	.87**	1					
V6	Fruit stalk length	−.45*	−.51*	−.55*	−.51*	−.51*	1				
V7	Fruit stalk weight	.70**	.59**	.75**	.68**	.67**	−.19	1			
V8	Titratable acidity	−.58**	.21	−.56**	−.49*	−.57**	−.12	.43	1		
V9	Total soluble solids	.70**	.22	.54*	.47*	.56**	.16	.17	−.23	1	
V10	Anthocyanin index	.45*	.33	.46*	.46*	.53*	−.15	.18	−.38	−.16	1

*, **: Correlation is significant at *p* ≤ .05 and .01 levels, respectively.

Fruit weight was considered as a dependent variable, and then the direct and indirect effects of each independent variable on this trait were calculated using stepwise regression analysis (MRA) (Table 3). Based on the results obtained, fruit weight showed significant *β* regression coefficients with fruit length and TSS, so that with the increase in both traits, the fruit weight increases.

**TABLE 3:** The traits associated with fruit weight in the studied sweet and sour cherries as revealed using MRA and coefficients.

Dependent trait	Independent trait	*r*	*r* ^*2*^	*β*	*t*	*p*
Fruit weight	Fruit length	.91 a	.84	.89	11.70	.00
	Total soluble solids	.95 b	.90	.26	3.45	.00

*Note:* The values in a column with different alphabetical letters are significantly different (*p* < .05).

PCA is one of the multivariate statistical methods used to identify important and influential traits. The purpose of PCA is to determine the number of important components to reduce the number of influential characters in the diagnosis and separation of genotypes. In addition, the confirmed relationships between traits by this method may be related to linkage relationships between trait‐controlling loci and phylotropic effects (Iezzoni & Pritts, 1991). Values higher than 0.65 were considered significant for each component. PCA results showed that 83.80% of the observed variance was explained by the first three components (Table 4). The first component (PC1) explained 53.92% of the total variance and included fruit weight, stone weight, fruit length, fruit width, fruit stalk weight, and anthocyanin index. The second component (PC2) explained 17.45% of the total variance and included fruit diameter and TA. The third component (PC3) explained 12.44% of the total variance and included fruit stalk length and TSS.

**TABLE 4:** Eigenvalues of the principal component axes from the PCA of the fruit characters in the studied sweet and sour cherries.

Trait	Component		
	1	2	3
Fruit weight	**0.95**	−0.14	0.15
Stone weight	**0.93**	0.29	0.01
Fruit length	**0.96**	−0.13	−0.08
Fruit width	**0.95**	0.09	0.08
Fruit diameter	0.18	**0.96**	0.07
Fruit stalk length	−0.59	0.03	**−0.65**
Fruit stalk weight	**0.75**	−0.13	0.20
Titratable acidity	0.47	**−0.76**	0.22
Total soluble solids	0.23	−0.06	**0.81**
Anthocyanin index	**0.76**	0.30	0.20
Total	5.39	1.75	1.24
% of variance	53.92	17.45	12.44
Cumulative %	53.92	71.37	83.80

*Note*: Bold values indicate the characteristics most influencing each PC.

**FIGURE 1:**
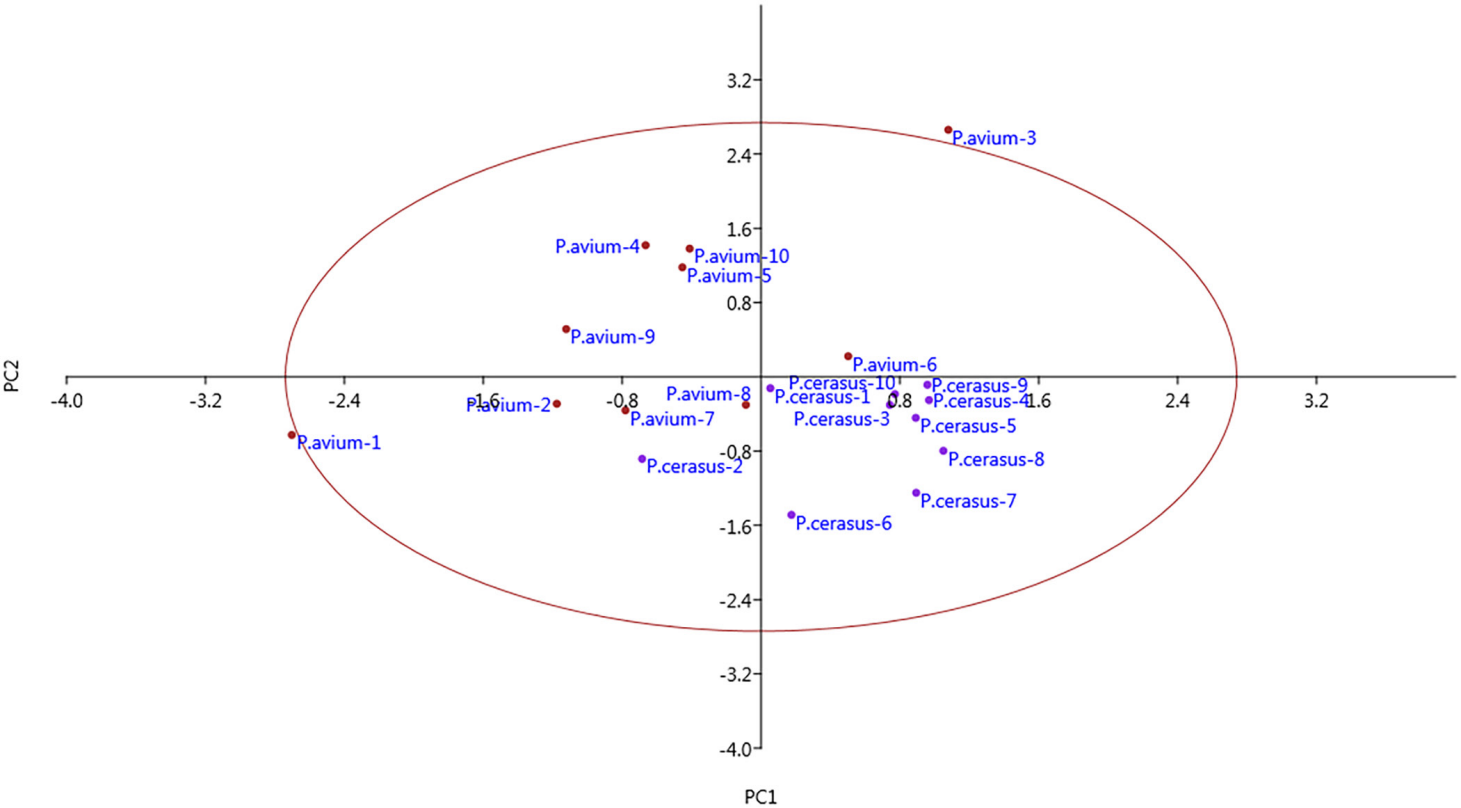
Biplot for the studied sweet and sour cherries based on PC1/PC2.

**FIGURE 2:**
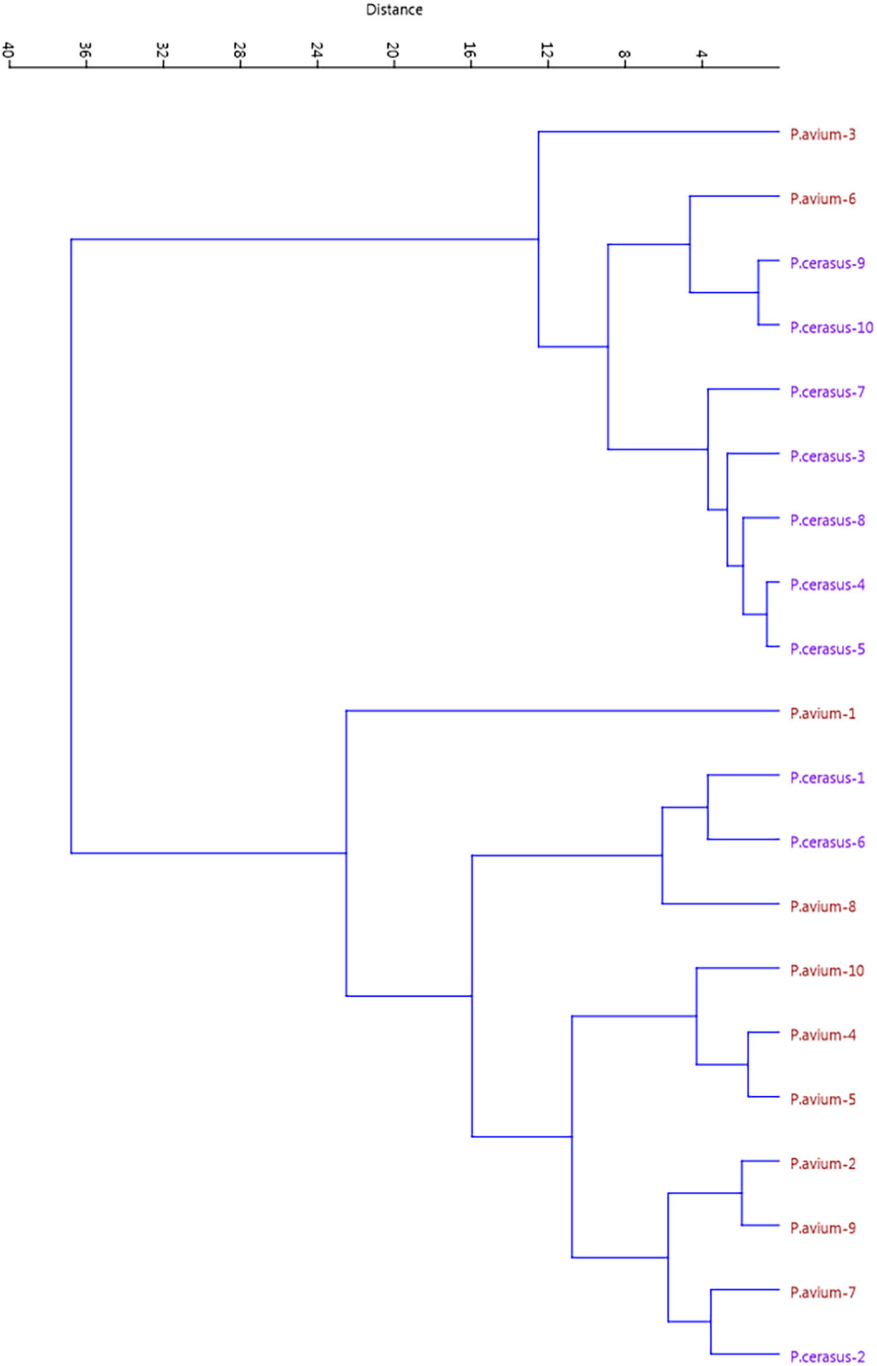
Ward cluster analysis of the studied sweet and sour cherries based on fruit traits using Euclidean distances.

Biplot analysis based on PC1 and PC2 separated genotypes of sweet and sour cherries from each other so that the genotypes were scattered on both sides of the biplot (Figure 1). If moving from positive values to negative values of PC1, a gradual increase in fruit weight, stone weight, fruit length, fruit width, fruit stalk weight, and anthocyanin index was observed. If moving from the negative side to the positive side of PC2, a gradual increase in fruit diameter and a gradual decrease in TA were observed. PCA provides a simple classification of these species for collection and modification. Also, the degree of similarity and dissimilarity was estimated using cluster analysis. The dendrogram was divided into two main clusters. The cluster analysis mostly separated genotypes of sweet and sour cherries and put them into two main groups, while two genotypes of sweet cherry were clustered with sour cherries, and three genotypes of sour cherry were clustered with sweet cherries (Figure 2). Rodrigues et al. (2008) reported that dendrogram obtained from morphological characteristics clearly separated sweet cherries from sour cherries. Also, Perez‐Sanchez et al. (2008) and Perez et al. (2010) reported that dendrogram gained from morphological parameters clearly showed differences between sweet cherries with sour and duke cherries. Description of morphological traits is a common and accepted method from a legal point of view to register and get points for cultivars (Khadivi et al., 2019; Khadivi‐Khub, 2014).

### 3.2 Molecular analysis

Four cpSSR primers, including TPScp1, TPScp2, TPScp3, and AB, produced distinct and different alleles among sweet and sour cherries (Table 5). These sites showed good transferability in the studied species and confirmed the integrity of the sequences of cpSSR sites in *Prunus* genus (Brettin et al., 2000).

**TABLE 5:** Locus names, number, and size of alleles detected cpSSRs used in studied sweet and sour cherries.

Locus	Sweet cherry		Sour cherry	
	Alleles observed (no.)	Product size range (bp)	Alleles observed (no.)	Product size range (bp)
TPScp1	1	193	1	192
TPScp2	1	108	1	151
TPScp3	1	206	1	205
AB	1	236	1	250

With TPScp1 locus, sweet cherry genotypes showed allele 193 bp, and sour cherry genotypes showed allele 192 bp. With TPScp2 locus, sweet cherry genotypes showed allele 108 bp, and sour cherry genotypes showed allele 151 bp. With TPScp3 locus, sweet cherry genotypes showed allele 206 bp and sour cherry genotypes showed allele 205 bp (Table 5). The observed polymorphism in TPScp3 sites was caused by the difference of 1–5 bp distance between alleles, which corresponds to a variable number of A or T base residues in the amplified region. This suggests that the observed variation is consistent with the stepwise mutation model (SMM), which can be explained by repetitive slippage at cpSSR loci (Valdes et al., 1993). In addition, the nonrecombining nature of the chloroplast genome means that unequal crossing over cannot be the main mechanism for this (Freimer & Slatkin, 1996).

With AB locus, sweet cherry genotypes showed allele 236 bp and sour cherry genotypes showed allele 250 bp (Table 5). Struss et al. (2002) investigated the chloroplast diversity of eight rootstocks of the Gisella group related to sweet cherry using the AB location, five of the rootstocks did not show alleles, and the other three produced two different alleles (238 and 249 bp). Ma et al. (2009) used four cpSSR loci to study four species of the *Cerasus* subgenus, which shared AB and TPScp1 primers in their study and the present study. In their species, the AB primer produced only the allele 251 bp and could not distinguish between their species, while in the present study, this locus produced two alleles, and interspecies variation was detected among sweet and sour cherries.

**FIGURE 3:**
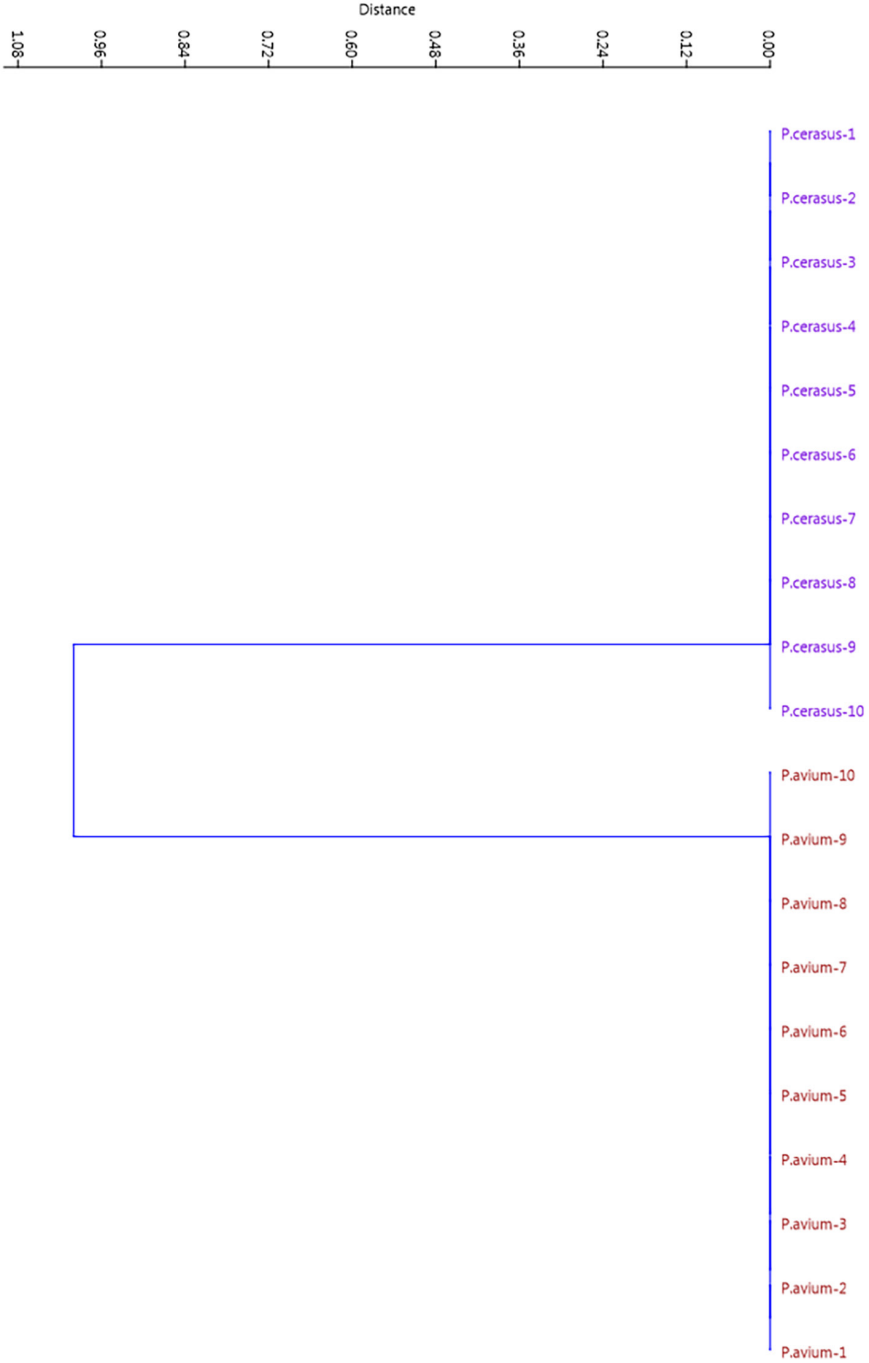
UPGMA cluster analysis of the studied sweet and sour cherries based on cpSSR data.

Chloroplast DNA is known to be a very conservative molecule; hence, the chances of detecting cpDNA polymorphism are low. But in the present study, the cpSSR loci separated sweet and sour cherries from each other (Figure 3), which confirms the theory that chloroplast genome of sour cherry (*P. cerasus*) is not derived from sweet cherry (*P. avium*) and is consistent with the studies of Olden and Nybom (1968) and Brettin et al. (2000). They reported that *P. avium* is not the maternal parent of *P. cerasus* and the chloroplast genome of *P. cerasus* is more similar to the chloroplast genome of ground cherry (*P. fruticosa*).

## 4 CONCLUSIONS

The knowledge of genetic diversity is essential for their survival, ecology, and development of appropriate germplasm for a diverse set of environments. The present results provided new insights regarding the extent of diversity of individuals and also determined the relatedness and obtained information on genetic diversity of sweet and sour cherries. The information provided here is useful for genetic improvement of cultivated cherries. The cpSSR loci separated sweet and sour cherries from each other, which confirms the theory that chloroplast genome of sour cherry (*P. cerasus*) is not derived from sweet cherry (*P. avium*). Overall, there was a high diversity in the germplasm, which could be exploited in further cultivation and improvement, and may be helpful for breeding programs and development of cultivars.

## AUTHOR CONTRIBUTIONS

**Ali Khadivi:** Formal analysis (lead); investigation (equal); methodology (lead); supervision (lead); writing – original draft (lead); writing – review and editing (lead). **Somayeh Goodarzi:** Investigation (equal). **Mostafakamal Shams:** Investigation (equal).

## CONFLICT OF INTEREST STATEMENT

The authors declare no conflicts of interest.

## Data Availability

The findings supporting the present study, when reasonable request, are available from the corresponding authors.

## References

[fsn33858-bib-0001] Ates, U. , & Ozturk, B. (2022). Fruit quality characteristics of different sweet cherry (*Prunus avium* L.) cultivars grown in Ordu province of Turkey. Karadeniz Fen Bilimleri Dergisi, 12(1), 168–177.

[fsn33858-bib-0002] Brettin, T. S. , Karle, R. , Crowe, E. L. , & Iezzoni, F. (2000). Chloroplast inheritance and DNA variation in sweet, sour and ground cherry. Heredity, 91, 74–79. 10.1093/jhered/91.1.75 10739133

[fsn33858-bib-0003] Brown, A. H. D. (1978). Isozymes, plant population genetic structure and genetic conservation. Theoretical and Applied Genetics, 52, 145–157. 24317500 10.1007/BF00282571

[fsn33858-bib-0004] Decroocq, V. , Hagen, L. S. , Favé, M. G. , Eyquard, J. P. , & Pierronnet, A. (2004). Microsatellite markers in the hexaploid *Prunus domestica* species and parentage lineage of three European plum cultivars using nuclear and chloroplast simple‐sequence repeats. Molecular Breeding, 13, 135–142.

[fsn33858-bib-0005] Doyle, J. J. , & Doyle, J. L. (1987). Isolation of DNA from fresh plant tissue. Focus, 12, 13–15.

[fsn33858-bib-0006] FAO STAT . 2021. http://www.faostat.fao.org

[fsn33858-bib-0007] Freimer, N. B. , & Slatkin, M. (1996). Microsatellites: Evolution and mutational processes. In Variation in the human genome (Vol. 197, pp. 51–72). Ciba foundation symposium. 10.1002/9780470514887.ch4 8827368

[fsn33858-bib-0008] Hammer, Ø. , Harper, D. A. T. , & Ryan, P. D. (2001). PAST: Paleontological statistics software package for education and data analysis. Palaeontologia Electronica, 4(1), 9.

[fsn33858-bib-0010] Hrotko, K. , Magyar, L. , & Gyeviki, M. (2008). Evaluation of native hybrids of *Prunus fruticosa* pall. as cherry interstocks. Acta Agriculturae Serbica, 25, 1341–1345.

[fsn33858-bib-0011] Iezzoni, A. F. , & Pritts, M. P. (1991). Applications of principal components analysis to horticultural research. HortScience, 26, 334–338.

[fsn33858-bib-0013] Khadivi, A. , Mohammadi, M. , & Asgari, M. K. (2019). Morphological and pomological characterizations of sweet cherry (*Prunus avium* L.), sour cherry (*Prunus cerasus* L.) and duke cherry (*Prunus × gondouinii* Rehd.) to choose the promising selections. Scientia Horticulturae, 257, 108719.

[fsn33858-bib-0014] Khadivi‐Khub, A. (2014). Assessment of cultivated cherry germplasm in Iran by multivariate analysis. Trees, 28, 669–685.

[fsn33858-bib-0015] Ma, H. , Olsen, R. , & Pooler, M. (2009). Evaluation of flowering cherry species, hybrids, and cultivars using simple sequence repeat markers. Journal of the American Society for Horticultural Science, 134(4), 435–444.

[fsn33858-bib-0016] Moreno, J. , & Manzano, M. A. (2002). Variedades de cerezo para el Valle del Jerte (p. 78). Junta de Extremadura Publishers.

[fsn33858-bib-0017] Norusis, M. J. (1998). SPSS/PC advanced statistics. SPSS Inc.

[fsn33858-bib-0018] Ohta, S. , Nishitani, C. , & Yamamoto, T. (2005). Chloroplast microsatellites in *Prunus*, Rosaseae. Molecular Ecology Notes, 5, 837–840.

[fsn33858-bib-0019] Ohta, S. , Osumi, S. , Katsuki, T. , Nakamura, I. , Yamamoto, T. , & Sato, Y. (2006). Genetic characterization of flowering cherries (*Prunus* subgenus *Cerasus*) using rpl16‐rpl14 spacer sequences of chloroplast DNA. Journal of the Japanese Society for Horticultural Science, 75, 72–78.

[fsn33858-bib-0020] Ohta, S. , Yamamoto, T. , Nishitani, C. , Katsuki, T. , & Iketani, H. (2007). Phylogenetic relationships among Japanese flowering cherries (*Prunus* subgenus *Cerasus*) based on nucleotide sequences of chloroplast DNA. Plant Systematics and Evolution, 263, 209–225.

[fsn33858-bib-0021] Olden, E. J. , & Nybom, N. (1968). On the origin of *Prunus cerasus* L. Hereditas, 59, 327–345.

[fsn33858-bib-0022] Perez, R. , Navarro, F. , Sanchez, M. A. , Ortiz, J. M. , & Morales, R. (2010). Analysis of agromorphological descriptors to differentiate between duke cherry (*Prunus × gondouinii* (Poit. & Turpin) Rehd.) and its progenitors: Sweet cherry (*Prunus avium* L.) and sour cherry (*Prunus cerasus* L.). Chilean Journal of Agricultural Research, 70, 34–49.

[fsn33858-bib-0023] Perez‐Sanchez, R. , Gomez‐Sanchez, M. A. , & Morales‐Corts, R. (2008). Agromorphological characterization of traditional Spanish sweet cherry (*Prunus avium* L.), sour cherry (*Prunus cerasus* L.) and duke cherry (*Prunus × gondouinii* Rehd.) cultivars. Spanish Journal of Agricultural Research, 6, 42–55.

[fsn33858-bib-0024] Rodrigues, L. C. , Morales, M. R. , Fernandes, A. J. B. , & Ortiz, J. M. (2008). Morphological characterization of sweet and sour cherry cultivars in a germplasm bank at Portugal. Genetic Resources and Crop Evolution, 55, 593–601.

[fsn33858-bib-0025] SAS Institute (Ed.). (1990). SAS® procedures version 6 (3rd ed.). SAS Institute.

[fsn33858-bib-0026] Struss, D. , Boritzki, M. , Karle, R. , & Iezzoni, A. F. (2002). Microsatellite markers differentiate eight Giessen cherry rootstocks. HortScience, 37, 191–193.

[fsn33858-bib-0027] Sugiura, M. (2003). History of chloroplast genomics. Photosynthesis Research, 76, 371–377. 16228593 10.1023/A:1024913304263

[fsn33858-bib-0028] Valdes, A. , Slatkin, M. , & Freimer, N. B. (1993). Allele frequencies at microsatellite loci: The stepwise mutation model revisited. Genetics, 133, 737–749. 8454213 10.1093/genetics/133.3.737 PMC1205356

[fsn33858-bib-0029] Webster, A. D. , & Looney, N. E. (1996). Cherries: Crop physiology and uses. CAB International Cambridge University Press.

[fsn33858-bib-0030] Xuan, H. , Neumueller, M. , & Schlottmann, P. Approaches to determine the origin of European plum (*Prunus domestica*) based on nucleotide sequences of chloroplast DNA. In: Proceedings of the 28th international horticultural congress, Lisboa, Portugal, 22–27 August. 2010.

